# BI-RADS 3–5 microcalcifications can preoperatively predict breast cancer HER2 and Luminal a molecular subtype

**DOI:** 10.18632/oncotarget.14655

**Published:** 2017-01-14

**Authors:** DongZhi Cen, Li Xu, Ningna Li, Zhiguang Chen, Lu Wang, Shuqin Zhou, Biao Xu, Chun ling Liu, Zaiyi Liu, Tingting Luo

**Affiliations:** ^1^ Department of Radiation Oncology and Department of Nuclear Medicine, The Third Affiliated Hospital of Guangzhou Medical University, Guangzhou, 510150, Guangdong Province, People's Republic of China; ^2^ Guangdong Provincial Traditional Chinese Medicine Hospital, Guangzhou, Guangdong Province 510120, P.R. China; ^3^ Department of Radiology, Guangdong General Hospital, Guangdong Academy of Medical Sciences, Guangzhou, Guangdong Province 510080, People's Republic of China; ^4^ Department of Ultrasound, The Third People's Hospital of Shenzhen, Guangdong Shenzhen 518112, China

**Keywords:** calcification, infiltrating ductal carcinoma, mammography, logistic regression, breast cancer molecular subtype

## Abstract

**Purpose:**

To investigate associations between breast cancer molecular subtype and the patterns of mammographically detected calcifications.

**Results:**

Identified were 93 (19.1%) Luminal A, 242 (49.9%) Luminal B, 108 (22.2%) HER2 and 42 (8.7%) basal subtypes. In univariate analysis, the clinicopathological parameters and BI-RADS 3–5 microcalcifications, which consisted 9 selected features was significantly associated with breast cancer molecular subtype (all *P* < 0.05). Among subtypes, multivariate analysis showed that calcification >2 cm in range (OR: 1.878, 95% CI: 1.150 to 3.067) and calcification > 0.5 mm in diameter (OR:2.206, 95% CI: 1.235 to 3.323) was independently predictive of HER2 subtype. The model showed good discrimination for predicting HER2 subtype, with a C-index of 0.704. In addition, multivariate analysis showed that calcification morphology (amorphour or coarse heterogenous calcifications OR: 2.847, 95% CI: 1.526 to 5.312) was independently predictive of Luminal A subtype. The model showed good discrimination for predicting Luminal A subtype, with a C-index of 0.74. And we demonstrated that amorphour or coarse heterogenous calcifications were associated with a higher incidence of Luminal A subtype than pleomorphic or fine linear or branching calcifications. There was no significant difference between breast cancer subtypes (Luminal B vs. other; Basal vs. other) and the patterns of mammographically detected calcifications.

**Materials and Methods:**

Mammographic images of 485 female patients were included. The correlation between mammographic imaging features and breast cancer subtype was analyzed using Chi-square test, univariate and binary logistic regression analysis.

**Conclusions:**

This study shows that BI-RADS 3–5 microcalcifications can be conveniently used to facilitate the preoperative prediction of HER2 and Luminal A molecular subtype in patients with infiltrating ductal carcinoma.

## INTRODUCTION

Worldwide, the most common invasive female cancer observed is breast cancer [1]. Molecular subtyping of breast cancer tissue samples has become a common practice for individualized disease management, elucidation of disease prognosis, and avoidance of overtreatment [2]. Breast cancer molecular subtypes based on immunohistochemical (IHC) markers include Luminal A/B, HER2, and basal-like [3]. Clinical differences have been recognized among these genetically distinct tumor. [3, 4] For example, relative to Luminal A-positive samples, HER-2–positive neoplasms were found to have nearly 2.0 times the likelihood of having four or more metastatic lymph nodes and 1.6 times the likelihood of having multifocal disease [3]. The characteristic features of Luminal A tumors include stage-1 disease, lymph node negativity, and well-differentiated cells, whereas those of Luminal B tumors include HER-2–positive nonluminal cancers with a high tumor grade, lymph-nodal metastases, robust proliferation, and advanced-stage disease [5].

Characteristic imaging phenotypes have been associated with the aforementioned three breast cancer molecular subtypes. The basal-like subtype has been associated with non-calcified, circumscribed masses with posterior acoustic enhancement. Luminal A/B subtype tumors are often spiculated masses with a poorly circumscribed margin. And HER2-enriched tumors have been observed to have pleomorphic calcifications [6]. Reported studies have used various factors to predict breast cancer molecular subtype such as genes associated with histopathologic features [7]; the MHC class II (MHC II) antigen presentation pathway [8]; features extracted from magnetic resonance images [9, 10, 11] and ultrasound features [2].

With the spread of screening mammography, microcalcifications have become a commonly observed positive sign of possible cancer [12]. The ability to predict molecular subtype reliably based on mammography findings would be helpful for treatment planning [13]. To the best of our knowledge, there is no literature that has determined whether a calcification features would enable superior prediction of breast cancer molecular subtype in invasive ductal carcinoma of breast. Therefore, the aim of this study was to investigate associations between breast cancer tumor molecular subtype and the patterns of mammographically detected calcifications.

## RESULTS

**Figure 1:**
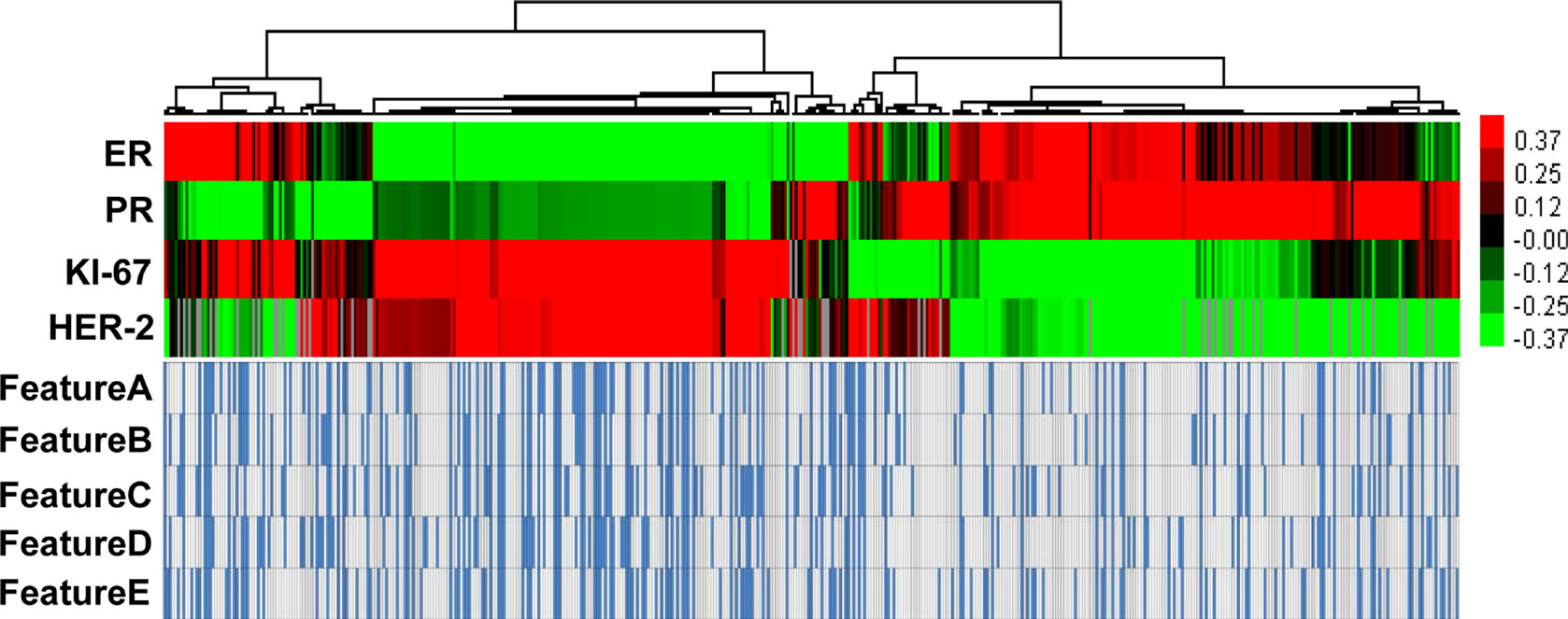
Hierarchical clustering yielded distinct groups of gene expression trends and patterns of mammographically detected calcifications

Hierarchical clustering yielded distinct groups of gene expression trends and patterns of mammographically detected calcifications (Figure 1). Breast cancers in the 485 patients were classified into molecular subtypes as follows: 93 (19.2%) Luminal A; 242 (49.9%) Luminal B; 108 (22.3%) HER2; and 42 (8.7%) Basal. In 485 patients, the average age was 51.7 ± 11.0 years (standard deviation). The average tumor size (from the pathology reports) was 2.1 cm ± 1.2. We graded the tumors as follows: 6.2% (30/485), low grade; 59.9% (264/441), intermediate grade; and 43.3% (191/441), high grade (Table 1).

**Table 1:** The patient and tumor characteristics per molecular subtype

	Molecular subtype				Pearson χ^2^ test	
	Luminal A	Luminal B	HER-2 enriched	Basal	χ^2^	*P* value
Age (years)					6.903	0.328†
< 35	7 (7.5)	17 (7.0)	3 (2.8)	3 (7.1)		
35–69	77 (82.8)	214 (88.4)	99 (91.7)	35 (83.3)		
≥ 70	9 (9.7)	11 (4.5)	6 (5.6)	4 (9.5)		
Grade					98.527	< 0.001†
grade 1	20 (21.5)	9 (3.7)	1 (0.9)	0 (0)		
grade 2	65 (69.9)	142 (58.7)	42 (38.9)	15 (25.7)		
grade 3	8 (8.6)	91 (37.6)	65 (60.2)	27 (64.3)		
Tumor Size					23.063	0.002 †
T1	59 (69.4)	112 (50.9)	36 (37.5)	17 (53.1)		
T2	26 (30.6)	104 (47.3)	55 (57.3)	15 (46.9)		
T3	0 (0)	4 (1.8)	5 (5.2)	0 ()		
Lymph node metastasis					8.852	0.031
Negative	45 (67.2)	98 (48.8)	52 (55.9)	20 (66.7)		
Positive	22 (32.8)	103 (51.2)	41 (44.1)	10 (33.3)		
Lymphovascular invasion					13.177	0.004
Negative	78 (86.7)	161 (69.4)	74 (71.8)	35 (85.4)		
Positive	12 (13.3)	71 (30.6)	29 (28,2)	6 (14.6)		
Feature A (Morphology)					23.531	< 0.001
Amorphour, Coarse heterogenous	80 (86.0)	173 (71.5)	61 (56.5)	34 (81.0)		
Pleomorphic, Fine linear or branching, Combined	13 (14.0)	69 (28.5)	47 (43.5)	8 (19.0)		
Feature B (Distribution)					15.618	0.001
Grouped or Clustered	79 (84.9)	170 (70.2)	65 (60.2)	32 (76.2)		
Linear, Segmental	14 (15.1)	72 (29.8)	43 (39.8)	10 (23.8)		
Feature C (Range)					20.149	< 0.001
< 2 cm	75 (80.6)	172 (71.1)	57 (52.8)	31 (73.8)		
≥ 2 cm	18 (19.4)	70 (28.9)	51 (47.2)	11 (26.2)		
Feature D (Diameter)					23.094	< 0.001
< 0.5 cm	78 (83.9)	180 (74.4)	59 (54.6)	29 (69.0)		
≥ 0.5 cm	15 (16.1)	62 (25.6)	49 (45.4)	13 (31.0)		
Feature E (Density)					11.858	< 0.001
< 20 cm^2^	71 (76.3)	165 (68.2)	60 (55.6)	32 (76.2)		
≥ 20 / cm^2^	22 (23.7)	77 (31.8)	48 (44.4)	10 (23.8)		

In univariate analysis, the clinicopathological parameters and BI-RADS 3–5 microcalcification categories, which consisted 9 selected features (grade: χ^2^ = 98.527; tumor size: χ^2^ = 23.063; lymph node metastasis: χ^2^ = 8.852; lymphovascular invasion: χ^2^ = 13.177; Feature A: χ^2^ = 23.531; Feature B: χ^2^ = 15.618; Feature C: χ^2^ = 20.149; Feature D: χ^2^ = 23.094; Feature E: χ^2^ = 11.858, Table 2), were associated with particular cancer molecular subtypes (all *P* < 0.05).

**Table 2:** The correlation between mammographic imaging features and breast cancer subtype(univariate logistic regression analysis)

		Luminal A		Luminal B		HER2		Basal	
		Odds ratio (95% CI)	Sig.	Odds ratio (95% CI)	Sig.	Odds ratio (95% CI)	Sig.	Odds ratio (95% CI)	Sig.
Feature A (Morphology)									
	Amorphour, Coarse heterogenous	2.847 (1.526,5.312)	0.001	Reference		Reference		Reference	
	Pleomorphic, Fine linear or branching, Combined	Reference		1.026 (0.691,1.524)	0.897	2.457 (1.570,3.846)	< 0.001	0.573 (0.258,1.271)	0.17
Feature B (Distribution)									
	Grouped or Clustered or Regional	Reference		Reference		Reference		Reference	
	Linear,Segmental	0.379 (0.206,0.694)	0.002	1.113 (0.750,1.650)	0.596	1.936 (1.235,3.036)	0.004	0.761 (0.363,1.593)	0.468
Feature C (Range)									
	< 2 cm	Reference		Reference		Reference		Reference	
	≥ 2 cm	0.473 (0.271,0.824)	0.008	0.829 (0.564,1.220)	0.341	2.512 (1.615,3.909)	< 0.001	0.776 (0.379,1.589)	0.488
Feature D (Diameter)									
	< 0.5 cm	Reference		Reference		Reference		Reference	
	≥ 0.5 cm	0.416 (0.230,0.751)	0.004	0.743 (0.500,1.103)	0.14	2.648 (1.694,4.140)	< 0.001	1.128 (0.568,2.239)	0.731
Feature E (Density)									
	< 20 cm2	Reference		Reference		Reference		Reference	
	≥ 20 / cm2	0.590 (0.350,0.994)	0.047	0.951 (0.650,1.391)	0.795	1.967 (1.267,3.054)	0.003	0.629 (0.301,1.315)	0.218

**Figure 2:**
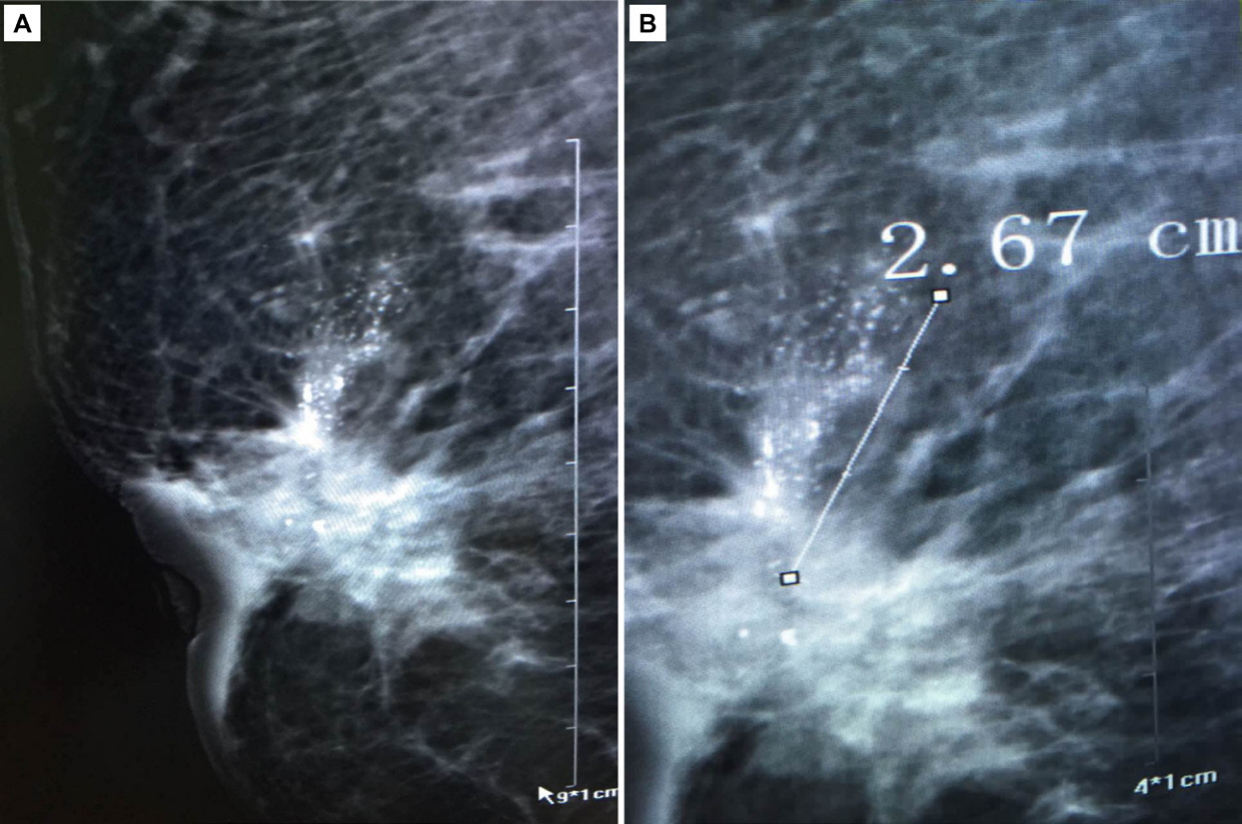
Infiltrating ductal carcinoma associated with microcalcification(Feature C:calcifications with > 2 cm in range and Feature D:calcifications with > 0.5 mm in diameter)

**Figure 3:**
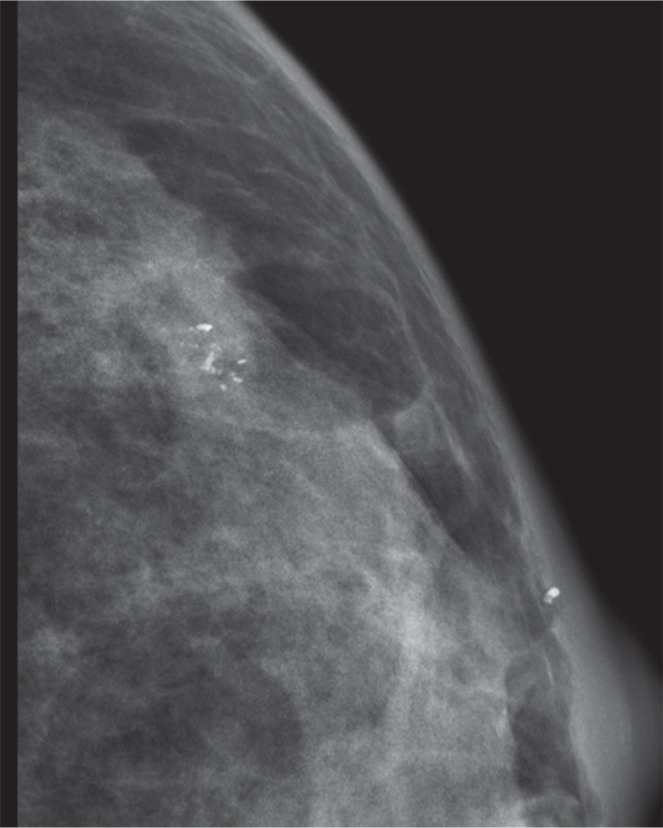
Infiltrating ductal carcinoma associated with microcalcification (Feature A: morphology— coarse heterogenous calcifications)

Multivariate analysis showed that calcification with a range > 2 cm (OR: 1.878 95% CI: 1.150–3.067) or calcification with a diameter > 0.5 mm (OR: 2.206 95% CI: 1.235–3.323) were predictive of the HER2 subtype (Figure 2). The model showed good discrimination for prediction of the HER2 subtype (C-index: 0.704). In addition, multivariate analysis showed that calcification morphology (amorphour or coarse heterogenous calcifications OR: 2.847 95% CI: 1.526–5.312; Figure 3) was independently predictive of Luminal A subtype (C-index: 0.74). And we demonstrated that amorphour or coarse heterogenous calcifications were associated with a higher incidence of Luminal A subtype than pleomorphic or fine linear or branching calcifications. We did not detect significant differences in imaged calcification patterns among the breast cancer subtypes (Luminal B vs. other; Basal vs. other). The results of the multivariate logistic regression analysis are shown in Table 3.

**Table 3:** the correlation between mammographic imaging features and breast cancer subtype (binary logistic regression analysis)

	Luminal A vs.other			HER2 vs.other		
	β	Odds ratio (95.0% C.I.)	Sig.	β	Odds ratio (95.0% C.I.)	Sig.
Feature A (Morphology)						
Amorphour, Coarse heterogenous	1.046	2.847 (1.526,5.312)	**0.001**			
Pleomorphic, Fine linear or branching, Combined	Reference					
Feature B (Distribution)						
Grouped or Clustered or Regional						
Linear,Segmental						
Feature C (Range)						
< 2 cm				Reference		
≥ 2 cm				0.63	1.878 (1.150,3.067)	**0.012**
Feature D (Diameter)						
< 0.5 cm				Reference		
≥ 0.5 cm				0.706	2.026 (1.235,3.323)	0.005
Feature E (Density)						
< 20 cm^2^						
≥ 20 / cm^2^						
Constant	−1.209			−1.719		

## DISCUSSION

Breast cancer is traditionally considered as a heterogeneous disease. Most breast biopsies are performed on masses that present in mammograms as a mass or microcalcification cluster [14]. Evaluation of observed calcifications is a major assessment parameter for mammographic images. Calcifications within breast tissue are a very early sign of *in situ* and IDC [15, 16]

In this study, we demonstrated associations between imaging features (related to clinicopathological parameters and BI-RADS 3–5 microcalcifications) and breast cancer molecular subtype. Finally, the multivariate logistic regression models show that HER-2 enriched molecular subtype is associated with calcifications with > 2 cm in range and calcifications with > 0.5 mm in diameter. In addition, multivariate analysis showed that calcification morphology (amorphour or coarse heterogenous calcifications OR: 2.847) was independently predictive of Luminal A subtype.

Amplification of *HER-2*(17q21-q22) or overexpression of the HER-2 protein are considered to have prognostic and therapeutic implications. Tumors of the HER-2 enriched subtype are often aggressive and recalcitrant to treatment [13]. Although fluorescent *in situ* hybridization is considered to be the gold standard for detection of HER-2 gene amplification in cases with ambiguous IHC, it presents a high cost barrier because of the specialized equipment and technical expertise needed to process the sample [17, 18].

Mammogram calcifications are more often associated with HER-2 overexpressing tumors than with non-HER-2 overexpressing tumors. For example, Seo et al. [19] found that calcifications were more frequent in tumors with HER2 overexpression than in those without it. Patel and coworkers [20] found that patients with tumors that overexpressed HER2 were more likely to have heterogeneous and pleomorphic calcifications. However, they did not measure the range, diameter, or density of the calcifications. The model showed good discrimination for predicting HER2 subtype, with a C-index of 0.704.

At the molecular level, Luminal A and B subtypes can be distinguished by the status of cell cycle-related and hormone-regulated pathways [21]. Luminal A breast cancers have better prognosis than other molecular subtypes. Luminal A cancers may also be insensitive to adjuvant chemotherapy [22]. Tamaki and coworkers [23] described several important factor divergences among luminal A tumors, such as: irregular and lobular versus round shape; speculated and indistinct versus microlobulated margins; amorphous versus pleomorphic calcification; and presence versus absence of cytoarchitectural distortion. In this study, we demonstrated that amorphous or coarse heterogenous calcifications were more likely to be Luminal A subtype tumor signs than were pleomorphic or fine linear or branching calcifications. The model showed good discrimination for predicting Luminal A subtype, with a C-index of 0.74.

This study has limitations that must be acknowledged. The main limitation was the sample size with only 42 patients in the basal subtype. This small sample size may have limited the power to detect additional correlations. A further study by using a larger pool of patients of basal subtype is required.

In conclusion, our findings clearly show that mammographic calcification features can be signs of breast cancer biological features. This study presents BI-RADS 3–5 microcalcifications can be conveniently used to facilitate the preoperative individualized prediction of HER2 and Luminal A molecular subtype in patients with infiltrating ductal carcinoma. This work provides useful information for pretreatment planning in breast cancer cases. Further work is needed to better define the relationships identified in our study and to explore additional relationships.

## MATERIALS AND METHODS

### Study subjects

The study was approved by our institutional review board, and written informed consent was obtained from all patients. Between January 2011 and April 2016, 485 consecutive patients who were referred for MG imaging of the breast and met the following inclusion criteria were respectively enrolled in our study for assessment: (1) Infiltrating ductal carcinoma (2) MG with intermediate-concern calcification or (3) MG calcifications with higher probability of malignancy. All clinical information was acquired through medical records.

### Mammography evaluation

Mammographic images were analyzed using a standard four view film. All images were reviewed by two radiologists, who had 12 (Liu CL) and 7 years (Xu L), respectively, of clinical experience in the interpretation of MG imaging for the patterns of mammographically detected calcifications; discrepancies were resolved by consensus. Calcifications were classified based on the BI-RADS classification system (Breast Imaging Reporting and Data System) lexicon [24, 25]. We conducted a detailed image analysis to evaluate the following features of the calcifications: morphology, distribution, range, diameter and density. Calcification morphology was divided into fine branching or casting, pleomorphic or combined; the distribution was classified as grouped or clustered, linear, segmental (Feature A, morphology; Feature B, distribution). We also performed some other measurements such as range, diameter and density to more comprehensively assess the appearance of these calcifications (Feature C, calcifications with ≤ 2 cm or > 2 cm in range; Feature D, ≤ 0.5 mm or > 0.5 mm in diameter; Feature E, ≤ 20 or > 20 per cm^2^ in density) [25].

### Breast cancer molecular subtypes

Four breast cancer molecular subtypes were classified by IHC based on previous reports: (1) the Luminal A subtype: ER and/or PR positive, and HER-2 negative, and Ki67 low < 14%; (2) the Luminal B subtype: ER and/or PR positive, and HER-2 negative, and Ki67 high ≥ 14% or ER and/or PR positive and HER-2 positive; (3) the HER-2 enriched group: HER-2 positive, and ER negative, and PR negative; and the basal subgroup: ER negative, PR negative, and HER-2 negative [26–28].

### Heat map diagram

Heat map was drawn to show a visual representation of gene expression trends. The amount of variation between different groups. The IHC expression of ER, PR, HER-2 and Ki-67 were plotted in a matrix by hierarchical clustering, performed by the Cluster v.3.0 program. Graphic outputs were generated by the Java TreeView v.1.6 software and presented in a color scale from green to red, where red indicated higher expression levels.

### Statistical analysis

We focused on the association of mammography imaging features mentioned above (Features A–E) with specific breast cancer molecular subtypes. The risk factors were evaluated by using Chi-square test, univariate and binary logistic regression analysis.

Binary logistic regression analysis was used separately for each breast cancer subtype. In this study breast cancer molecular subtype was a binary variable (1 when a tumor belonged to the breast cancer subtype of interest, such as HER-2 enriched; and 0 if it belonged to any other molecular subtype). The logistic regression was repeated for the four breast cancer molecular subtypes: Luminal A, Luminal B, HER2 and Basal. The multivariate logistic regression models were constructed by using the binary logistic regression function in the SPSS statistical software package (version 15.0; SPSS Company, Chicago, IL). Then, the association of the imaging variables (Feature A–E) with each specific subtype was evaluated by using the likelihood ratio test function in SPSS statistical software package. Due to the small imaging variables, we included all the imaging variables (Features A–E). Discrimination was measured with the concordance index, similar to the area under the receiver operating characteristic curve: values range from 0.5 (nodiscrimination) to 1.0 (perfect discrimination) [29, 30].

## Abbrivations

Features A: Amorphour, Coarse heterogenous Pleomorphic, Fine linear or branching.
Features B: Grouped or clustered Linear, Segmental
Features C: Calcifications ≤ 2 cm in range Calcifications > 2 cm in range.
Features D: Calcifications ≤ 0.5 cm in diameter Calcifications > 0.5 cm in diameter.
Features E: Calcifications ≤ 20/cm^2^ in density Calcifications ≤ 20/cm^2^ in density.
